# Supplementary material for: A Likelihood Approach for Real-Time Calibration of Stochastic Compartmental Epidemic Models
Source: PLoS Comput Biol. 2017 Jan 17;13(1):e1005257. doi: 10.1371/journal.pcbi.1005257 (PMC5240920; doi:10.1371/journal.pcbi.1005257)
Supplement: S1 File — (TAR.GZ) [file pcbi.1005257.s014.tar.gz › HSPH_Online-SI-Revision/EnKF07/n1-mild/n1-mild_EnKF2_table-8.pdf]

1	newly 3 weeks spec	14.	39.66	{2., 70.}	219.429	0.53863	newly 3 weeks cumul	37.	296.63	{82., 699.}	708.838	0.788143	newly 5 weeks spec	24.	11.95	{0., 28.}	78.2083	0.973309	newly 5 weeks cumul	75.	330.75	{100., 742.}	349.213	0.550551	newly 63 weeks cumul	382.	360.25	{120., 781.}	61.4319	0.311875
2	newly 3 weeks spec	27.	12.58	{0., 20.}	95.5556	1.03424	newly 3 weeks cumul	86.	95.97	{13., 159.}	108.849	0.479624	newly 5 weeks spec	28.	5.27	{0., 10.}	91.3214	1.58213	newly 5 weeks cumul	143.	108.83	{15., 189.}	98.2448	0.601187	newly 59 weeks cumul	317.	119.07	{15., 264.}	88.5584	0.851535
3	newly 3 weeks spec	15.	46.83	{1., 117.}	255.933	0.627396	newly 3 weeks cumul	45.	270.92	{20., 741.}	519.422	0.639044	newly 5 weeks spec	22.	31.76	{0., 100.}	142.818	1.0703	newly 5 weeks cumul	87.	339.9	{41., 743.}	315.264	0.560289	newly 68 weeks cumul	303.	487.24	{60., 911.}	110.033	0.453978
4	newly 3 weeks spec	11.	24.59	{2., 56.}	166.818	0.446794	newly 3 weeks cumul	28.	202.06	{49., 400.}	623.357	0.69355	newly 5 weeks spec	15.	7.45	{0., 19.}	93.4	1.13648	newly 5 weeks cumul	54.	222.28	{56., 485.}	317.815	0.478626	newly 62 weeks cumul	437.	242.94	{56., 567.}	71.0114	0.516225
5	newly 3 weeks spec	17.	50.08	{3., 113.}	227.176	0.561922	newly 3 weeks cumul	65.	360.58	{120., 853.}	458.154	0.660886	newly 5 weeks spec	31.	16.65	{0., 40.}	72.0968	0.925903	newly 5 weeks cumul	108.	406.2	{146., 920.}	280.407	0.509621	newly 64 weeks cumul	446.	431.5	{155., 935.}	51.9955	0.266049
6	newly 3 weeks spec	1.	43.33	{1., 120.}	4249.	1.34007	newly 3 weeks cumul	6.	310.48	{19., 825.}	5081.33	1.44543	newly 5 weeks spec	7.	29.24	{0., 96.}	402.857	1.05538	newly 5 weeks cumul	16.	372.87	{24., 828.}	2239.81	1.18261	newly 68 weeks cumul	409.	485.15	{35., 917.}	77.7188	0.488189
7	newly 3 weeks spec	31.	89.2	{2., 195.}	231.355	0.645661	newly 3 weeks cumul	65.	442.2	{57., 830.}	589.477	0.752657	newly 5 weeks spec	13.	42.08	{0., 110.}	278.769	0.826881	newly 5 weeks cumul	100.	547.56	{86., 895.}	457.68	0.686358	newly 67 weeks cumul	468.	635.38	{159., 1050.}	68.453	0.302668
8	newly 3 weeks spec	7.	21.75	{0., 59.}	287.286	0.613469	newly 3 weeks cumul	20.	131.45	{15., 354.}	569.55	0.494632	newly 5 weeks spec	14.	6.47	{0., 13.}	102.357	1.37419	newly 5 weeks cumul	41.	149.09	{17., 442.}	295.732	0.375155	newly 59 weeks cumul	476.	159.63	{18., 453.}	88.4895	0.976905
9	newly 3 weeks spec	17.	9.6	{0., 16.}	113.176	1.48159	newly 3 weeks cumul	37.	107.41	{5., 241.}	246.459	0.443551	newly 5 weeks spec	20.	3.09	{0., 6.}	99.15	1.87757	newly 5 weeks cumul	77.	115.66	{5., 267.}	153.351	0.594731	newly 58 weeks cumul	379.	120.82	{5., 270.}	94.0528	1.11691
10	newly 3 weeks spec	34.	14.95	{0., 20.}	103.618	1.1876	newly 3 weeks cumul	83.	109.14	{12., 246.}	133.855	0.548677	newly 5 weeks spec	34.	3.91	{0., 8.}	90.3235	1.78854	newly 5 weeks cumul	149.	120.13	{14., 300.}	110.141	0.704545	newly 59 weeks cumul	381.	124.53	{14., 337.}	90.7585	1.00614
11	newly 3 weeks spec	5.	6.1	{0., 13.}	143.2	1.01268	newly 3 weeks cumul	14.	66.33	{6., 72.}	397.357	0.485131	newly 5 weeks spec	14.	2.13	{0., 4.}	94.9286	1.7369	newly 5 weeks cumul	35.	71.79	{6., 86.}	160.143	0.401926	newly 58 weeks cumul	476.	76.53	{6., 99.}	90.0147	1.21125
12	newly 3 weeks spec	4.	21.81	{0., 57.}	509.25	0.706378	newly 3 weeks cumul	11.	154.61	{13., 468.}	1315.55	0.724185	newly 5 weeks spec	8.	5.89	{0., 15.}	115.125	1.19517	newly 5 weeks cumul	23.	171.6	{13., 520.}	667.304	0.540411	newly 59 weeks cumul	336.	179.82	{13., 520.}	95.5417	0.906765
13	newly 3 weeks spec	29.	28.77	{0., 113.}	131.621	1.01683	newly 3 weeks cumul	92.	184.03	{18., 683.}	170.967	0.486779	newly 5 weeks spec	28.	10.37	{0., 29.}	100.393	1.53699	newly 5 weeks cumul	149.	212.1	{21., 828.}	136.604	0.571532	newly 60 weeks cumul	314.	229.68	{22., 903.}	97.4459	0.743573
14	newly 3 weeks spec	28.	52.82	{1., 139.}	133.	0.50901	newly 3 weeks cumul	69.	381.1	{133., 909.}	457.217	0.657199	newly 5 weeks spec	32.	18.	{0., 45.}	76.25	0.93875	newly 5 weeks cumul	123.	429.65	{142., 1012.}	255.26	0.47965	newly 64 weeks cumul	351.	458.4	{158., 1065.}	67.7094	0.265238
15	newly 3 weeks spec	16.	52.59	{6., 119.}	254.688	0.582002	newly 3 weeks cumul	45.	364.78	{136., 852.}	713.556	0.820896	newly 5 weeks spec	24.	17.	{0., 43.}	74.75	0.799957	newly 5 weeks cumul	89.	411.41	{167., 931.}	365.989	0.595194	newly 64 weeks cumul	454.	437.21	{176., 941.}	51.0815	0.256632
16	newly 3 weeks spec	21.	30.7	{2., 72.}	122.762	0.524942	newly 3 weeks cumul	39.	211.35	{36., 518.}	453.667	0.594693	newly 5 weeks spec	16.	10.14	{0., 23.}	98.375	1.13527	newly 5 weeks cumul	78.	238.	{36., 590.}	228.846	0.438383	newly 62 weeks cumul	411.	265.69	{36., 708.}	74.4209	0.565884
17	newly 3 weeks spec	8.	53.56	{5., 122.}	583.75	0.738589	newly 3 weeks cumul	19.	525.29	{175., 960.}	2664.68	1.32787	newly 5 weeks spec	11.	16.96	{0., 48.}	151.455	0.946596	newly 5 weeks cumul	37.	569.93	{234., 1031.}	1440.51	1.08803	newly 65 weeks cumul	358.	604.85	{234., 1072.}	88.9637	0.277628
18	newly 3 weeks spec	10.	9.71	{0., 16.}	133.3	1.1671	newly 3 weeks cumul	30.	102.84	{4., 124.}	290.6	0.444158	newly 5 weeks spec	13.	2.93	{0., 5.}	99.4615	1.6877	newly 5 weeks cumul	62.	111.43	{4., 138.}	162.984	0.503133	newly 58 weeks cumul	369.	115.38	{4., 142.}	94.8401	1.09604
19	newly 3 weeks spec	26.	52.7	{5., 112.}	140.385	0.461347	newly 3 weeks cumul	62.	521.27	{176., 971.}	743.919	0.831967	newly 5 weeks spec	40.	15.94	{0., 46.}	78.8	1.16643	newly 5 weeks cumul	140.	564.23	{236., 1048.}	309.521	0.552913	newly 65 weeks cumul	489.	599.35	{237., 1073.}	56.8691	0.237121
20	newly 3 weeks spec	15.	25.25	{2., 47.}	130.6	0.469178	newly 3 weeks cumul	42.	188.59	{36., 448.}	360.452	0.516751	newly 5 weeks spec	12.	8.44	{0., 29.}	100.667	1.01302	newly 5 weeks cumul	79.	211.07	{37., 474.}	190.747	0.386371	newly 62 weeks cumul	337.	235.33	{38., 644.}	72.727	0.496014
21	newly 3 weeks spec	3.	26.98	{1., 46.}	826.	0.755605	newly 3 weeks cumul	24.	225.	{19., 523.}	851.667	0.809637	newly 5 weeks spec	14.	8.56	{0., 22.}	102.857	1.21511	newly 5 weeks cumul	48.	247.82	{21., 566.}	435.708	0.612998	newly 62 weeks cumul	267.	270.68	{21., 713.}	85.1461	0.486662
22	newly 3 weeks spec	11.	54.66	{3., 127.}	424.909	0.710182	newly 3 weeks cumul	21.	385.85	{153., 870.}	1737.67	1.15904	newly 5 weeks spec	8.	17.98	{0., 44.}	190.25	0.79206	newly 5 weeks cumul	39.	435.85	{168., 969.}	1018.9	0.957252	newly 64 weeks cumul	470.	462.38	{172., 983.}	49.8255	0.246151
23	newly 3 weeks spec	17.	17.04	{0., 24.}	126.824	0.81759	newly 3 weeks cumul	40.	111.24	{11., 324.}	219.6	0.356096	newly 5 weeks spec	12.	4.75	{0., 11.}	96.9167	1.29299	newly 5 weeks cumul	68.	124.86	{15., 355.}	155.647	0.407536	newly 59 weeks cumul	373.	131.07	{15., 355.}	87.8954	0.905192
24	newly 3 weeks spec	11.	8.41	{0., 20.}	84.6364	0.77976	newly 3 weeks cumul	30.	172.3	{15., 443.}	492.533	0.62451	newly 5 weeks spec	19.	1.61	{0., 3.}	97.9474	1.86089	newly 5 weeks cumul	65.	176.94	{15., 449.}	204.431	0.445231	newly 61 weeks cumul	445.	178.52	{15., 449.}	72.8315	0.70942
25	newly 3 weeks spec	8.	8.44	{0., 10.}	138.75	1.14162	newly 3 weeks cumul	23.	104.78	{7., 105.}	382.609	0.460851	newly 5 weeks spec	6.	3.35	{0., 6.}	114.167	1.34866	newly 5 weeks cumul	43.	112.87	{7., 107.}	209.698	0.399401	newly 58 weeks cumul	330.	117.37	{7., 107.}	93.6333	0.982301
26	newly 3 weeks spec	19.	11.23	{0., 22.}	85.5263	0.928445	newly 3 weeks cumul	55.	131.63	{14., 329.}	172.345	0.435175	newly 5 weeks spec	16.	2.36	{0., 6.}	91.875	1.7026	newly 5 weeks cumul	94.	139.05	{14., 369.}	95.4574	0.371419	newly 61 weeks cumul	295.	141.47	{14., 369.}	71.3458	0.633248
27	newly 3 weeks spec	21.	7.7	{0., 14.}	103.333	1.62021	newly 3 weeks cumul	42.	90.03	{8., 95.}	174.881	0.388982	newly 5 weeks spec	24.	2.7	{0., 4.}	98.0833	2.05824	newly 5 weeks cumul	84.	97.07	{8., 111.}	122.893	0.556226	newly 58 weeks cumul	327.	100.7	{8., 117.}	94.4037	1.03675
28	newly 3 weeks spec	3.	52.93	{3., 117.}	1679.	1.0578	newly 3 weeks cumul	9.	353.75	{137., 751.}	3830.56	1.49324	newly 5 weeks spec	4.	17.59	{0., 42.}	402.75	0.905068	newly 5 weeks cumul	17.	401.21	{153., 886.}	2260.53	1.27307	newly 64 weeks cumul	356.	426.21	{155., 916.}	56.0702	0.238734
29	newly 3 weeks spec	18.	9.32	{0., 20.}	79.1111	0.882238	newly 3 weeks cumul	43.	161.74	{16., 429.}	299.86	0.509451	newly 5 weeks spec	35.	1.32	{0., 3.}	96.2286	2.10758	newly 5 weeks cumul	101.	166.77	{16., 438.}	112.901	0.378979	newly 61 weeks cumul	354.	168.	{16., 439.}	71.791	0.648687
30	newly 3 weeks spec	14.	57.65	{5., 126.}	335.357	0.611925	newly 3 weeks cumul	49.	532.93	{167., 967.}	989.816	0.938991	newly 5 weeks spec	21.	18.09	{0., 48.}	94.619	0.964729	newly 5 weeks cumul	98.	580.95	{232., 1043.}	497.051	0.705378	newly 65 weeks cumul	412.	618.39	{235., 1061.}	73.2985	0.254486
31	newly 3 weeks spec	26.	38.53	{1., 74.}	108.885	0.489108	newly 3 weeks cumul	72.	311.69	{88., 626.}	337.819	0.533303	newly 5 weeks spec	44.	12.	{0., 32.}	81.5455	1.19634	newly 5 weeks cumul	142.	344.14	{104., 730.}	154.366	0.329964	newly 63 weeks cumul	451.	361.64	{119., 815.}	59.082	0.333478
32	newly 3 weeks spec	6.	10.6	{0., 24.}	148.667	0.637376	newly 3 weeks cumul	17.	152.	{18., 388.}	801.647	0.759859	newly 5 weeks spec	8.	2.64	{0., 5.}	97.	1.35206	newly 5 weeks cumul	37.	159.2	{18., 403.}	350.595	0.541903	newly 61 weeks cumul	420.	162.15	{18., 423.}	73.0833	0.712378
33	newly 3 weeks spec	16.	29.21	{2., 59.}	141.938	0.471182	newly 3 weeks cumul	37.	198.3	{36., 443.}	447.081	0.578294	newly 5 weeks spec	21.	10.22	{0., 21.}	88.4762	1.0388	newly 5 weeks cumul	77.	224.6	{39., 477.}	215.61	0.413035	newly 62 weeks cumul	388.	254.02	{39., 648.}	72.5722	0.529849
34	newly 3 weeks spec	7.	9.48	{0., 26.}	114.286	0.671022	newly 3 weeks cumul	27.	142.68	{13., 344.}	447.185	0.638234	newly 5 weeks spec	14.	2.46	{0., 5.}	97.8571	1.71196	newly 5 weeks cumul	53.	149.52	{13., 377.}	212.604	0.487818	newly 61 weeks cumul	269.	155.4	{13., 425.}	71.8067	0.607644
35	newly 3 weeks spec	10.	42.47	{1., 72.}	356.1	0.634355	newly 3 weeks cumul	37.	305.82	{88., 799.}	733.189	0.806189	newly 5 weeks spec	22.	12.71	{0., 35.}	81.8636	0.949164	newly 5 weeks cumul	79.	341.47	{102., 823.}	340.215	0.549779	newly 63 weeks cumul	462.	363.21	{116., 859.}	60.7597	0.366969
36	newly 3 weeks spec	18.	61.35	{6., 137.}	272.167	0.591415	newly 3 weeks cumul	39.	516.07	{108., 957.}	1225.15	1.01138	newly 5 weeks spec	49.	20.92	{0., 56.}	76.2041	1.11047	newly 5 weeks cumul	114.	570.85	{130., 1066.}	406.149	0.632133	newly 65 weeks cumul	404.	614.93	{170., 1132.}	76.2252	0.262511
37	newly 3 weeks spec	12.	9.7	{0., 23.}	80.3333	0.724847	newly 3 weeks cumul	26.	155.64	{18., 421.}	511.846	0.629323	newly 5 weeks spec	13.	2.7	{0., 5.}	98.1538	1.58865	newly 5 weeks cumul	55.	162.77	{18., 427.}	221.545	0.442006	newly 61 weeks cumul	310.	168.74	{18., 450.}	71.5355	0.584177
38	newly 3 weeks spec	35.	56.23	{4., 110.}	110.6	0.492452	newly 3 weeks cumul	65.	505.12	{118., 952.}	680.615	0.79532	newly 5 weeks spec	24.	19.2	{0., 48.}	91.5	0.967555	newly 5 weeks cumul	117.	556.22	{141., 1054.}	380.906	0.612098	newly 65 weeks cumul	464.	599.46	{185., 1110.}	61.0603	0.245958
39	newly 3 weeks spec	28.	42.99	{4., 79.}	114.679	0.500689	newly 3 weeks cumul	72.	275.59	{76., 702.}	291.431	0.478679	newly 5 weeks spec	31.	14.83	{0., 40.}	76.871	0.940101	newly 5 weeks cumul	130.	313.97	{92., 830.}	157.5	0.336804	newly 63 weeks cumul	377.	341.33	{101., 830.}	65.2971	0.338175
40	newly 3 weeks spec	16.	99.36	{16., 214.}	535.375	0.746528	newly 3 weeks cumul	56.	494.91	{166., 877.}	790.946	0.884125	newly 5 weeks spec	15.	42.46	{0., 91.}	226.933	0.687029	newly 5 weeks cumul	83.	602.04	{240., 1037.}	631.494	0.817423	newly 66 weeks cumul	397.	703.54	{304., 1134.}	90.1058	0.295165
41	newly 3 weeks spec	13.	87.4	{1., 184.}	598.	0.86659	newly 3 weeks cumul	28.	428.23	{94., 842.}	1433.89	1.08006	newly 5 weeks spec	36.	47.93	{0., 117.}	98.8056	0.70975	newly 5 weeks cumul	89.	542.72	{118., 927.}	516.27	0.731528	newly 67 weeks cumul	337.	659.55	{198., 1044.}	112.448	0.344101
42	newly 3 weeks spec	5.	93.76	{12., 213.}	1780.4	1.11244	newly 3 weeks cumul	20.	486.49	{164., 905.}	2335.45	1.27307	newly 5 weeks spec	30.	39.85	{0., 87.}	96.9	0.650839	newly 5 weeks cumul	64.	585.57	{228., 1055.}	820.391	0.898422	newly 66 weeks cumul	325.	672.83	{275., 1122.}	119.129	0.348959
43	newly 3 weeks spec	39.	42.67	{1., 113.}	90.7949	0.642596	newly 3 weeks cumul	61.	297.98	{23., 774.}	410.393	0.595026	newly 5 weeks spec	44.	30.67	{0., 103.}	90.0682	1.19122	newly 5 weeks cumul	146.	363.97	{37., 774.}	186.815	0.499588	newly 68 weeks cumul	494.	500.02	{53., 916.}	62.3887	0.425161
44	newly 3 weeks spec	19.	17.44	{0., 40.}	119.684	1.02755	newly 3 weeks cumul	73.	146.14	{14., 331.}	157.918	0.387587	newly 5 weeks spec	14.	6.53	{0., 13.}	105.929	1.39128	newly 5 weeks cumul	103.	162.69	{15., 418.}	134.282	0.43937	newly 60 weeks cumul	300.	175.68	{15., 474.}	89.2533	0.714087
45	newly 3 weeks spec	26.	8.17	{0., 12.}	100.115	1.48222	newly 3 weeks cumul	61.	82.83	{6., 99.}	117.	0.453168	newly 5 weeks spec	23.	2.71	{0., 3.}	99.7826	1.9628	newly 5 weeks cumul	105.	89.79	{6., 107.}	101.914	0.606989	newly 58 weeks cumul	383.	96.73	{6., 110.}	91.564	1.07045
46	newly 3 weeks spec	13.	55.01	{5., 118.}	344.692	0.621115	newly 3 weeks cumul	26.	515.57	{117., 991.}	1883.35	1.17817	newly 5 weeks spec	17.	17.1	{0., 48.}	106.	0.845249	newly 5 weeks cumul	55.	561.05	{126., 1059.}	922.091	0.913333	newly 65 weeks cumul	489.	597.43	{162., 1075.}	60.2679	0.251449
47	newly 3 weeks spec	7.	42.2	{3., 76.}	527.714	0.736463	newly 3 weeks cumul	19.	298.39	{83., 623.}	1477.11	1.06867	newly 5 weeks spec	6.	14.45	{0., 34.}	225.167	0.847306	newly 5 weeks cumul	28.	337.32	{97., 811.}	1112.07	0.965462	newly 63 weeks cumul	357.	364.13	{106., 873.}	66.0308	0.357538
48	newly 3 weeks spec	17.	6.69	{0., 14.}	90.5294	1.24071	newly 3 weeks cumul	40.	104.47	{9., 261.}	205.225	0.374718	newly 5 weeks spec	17.	2.85	{0., 6.}	96.6471	1.70823	newly 5 weeks cumul	72.	111.84	{9., 265.}	135.083	0.450945	newly 58 weeks cumul	326.	118.97	{9., 265.}	88.9601	0.913222
49	newly 3 weeks spec	2.	24.68	{4., 50.}	1141.	0.890631	newly 3 weeks cumul	12.	189.92	{43., 315.}	1483.5	1.02954	newly 5 weeks spec	8.	8.67	{0., 22.}	119.375	0.845916	newly 5 weeks cumul	27.	212.59	{54., 354.}	689.667	0.740516	newly 62 weeks cumul	311.	240.7	{54., 535.}	69.8971	0.409336
50	newly 3 weeks spec	19.	17.05	{0., 27.}	121.	0.915453	newly 3 weeks cumul	64.	95.02	{7., 186.}	134.281	0.476215	newly 5 weeks spec	20.	6.14	{0., 9.}	97.8	1.47546	newly 5 weeks cumul	105.	111.01	{8., 271.}	115.952	0.584921	newly 59 weeks cumul	406.	119.95	{8., 306.}	88.2586	1.01309
